# Effectiveness of Meaning-Centered Coaching on the Job of Oncology Nurses on Spiritual Care Competences: A Participatory Action Research Approach

**DOI:** 10.1097/NCC.0000000000001255

**Published:** 2023-07-04

**Authors:** Linda Modderkolk, Jacqueline van Meurs, Veronique de Klein, Yvonne Engels, Anne B. Wichmann

**Affiliations:** Author Affiliations: Department of Anaesthesiology, Pain and Palliative Medicine (Modderkolk, Engels and Wichmann), Department of Primary and Community Care (Modderkolk), Spiritual Care and Pastoral Service (van Meurs), Department of Medical Oncology (de Klein), Radboudumc, Nijmegen, the Netherlands.

**Keywords:** Clinical trial, Coaching, Communication, Oncology nursing, Patient-centered care, Spirituality

## Abstract

**Background:**

Nurses’ competences in providing spiritual care can increase quality of care for and quality of life of patients with cancer and job satisfaction but are often suboptimal. Training to improve this mostly takes place off-site, although implementation in daily care practice is key.

**Objectives:**

The aims of this study were to implement a meaning-centered coaching on the job intervention and to measure its effects on oncology nurses’ spiritual care competences and job satisfaction, and factors influencing this.

**Methods:**

A participatory action research approach was adopted. Mixed methods were used to assess intervention effects in which nurses of an oncology ward in a Dutch academic hospital participated. Spiritual care competences and job satisfaction were quantitatively measured and complemented with content analysis of qualitative data.

**Results:**

Thirty nurses participated. A significant increase in spiritual care competences was found, particularly regarding communication, personal support, and professionalization. More self-reported awareness of personal experiences in caring for patients, and an increase in mutual communication and involvement around meaning-centered care provision as a team were found. Mediating factors were related to nurses’ attitudes, support structures, and professional relations. No significant impact was found on job satisfaction.

**Conclusion:**

Meaning-centered coaching on the job increased oncology nurses’ spiritual care competences. Nurses developed a more exploratory attitude in their communication with patients—instead of acting based on their own assumptions about what is of meaning.

**Implications for Practice:**

Attention to and improving spiritual care competences should be integrated into existing work structures, and terminology used should match existing understandings and sentiments.

Receiving a cancer diagnosis can have a huge impact on a person’s life: on both a practical and a more profound level.^1–4^ Although cancer treatment is often highly protocolized, a patient’s life context—ranging from the financial situation to the social system, personal values, and meaning—often is of such importance that it impacts or should impact treatment preferences and decision making.^5,6^ Consequently, not integrating it could compromise both quality of life and quality of care.^6^ An essential part of personalized, contextual care is the spiritual dimension. It regards “the dynamic dimension of human life that relates to the way persons (individuals and community) experience, express and/or seek meaning, purpose and transcendence, and the way they connect to the moment, to self, to others, to nature, to the significant and/or the sacred”^7^ and has a multidimensional nature—consisting of existential challenges, value-based considerations and attitudes, and religious considerations and foundations.^8^ Identifying and exploring aspects related to this dimension can be performed in any encounter between patient and healthcare provider and should start at the time of diagnosis and continue through to end of life or survivorship.^9^ However, specifically in the oncology ward, nurses spend time with hospitalized patients with cancer, giving them a well-placed and important role in providing it.^10^

A prerequisite for spiritual care provision is having the communication skills to identify and explore what is of meaning for a patient. Communication is a central element in daily nursing practice, and several studies showed the effects of enhancing communication between nurses and patients in oncology care. It not only can improve patients’ physical and mental well-being but also seems to be a vital source of nurses’ job satisfaction.^10–12^ However, addressing the spiritual dimension during communication seems to be challenging.^10,13^ In practice, this leads to nurses underestimating its importance, a lack of knowledge on the topics addressed by the patient, and limited skills to explore them.^14^ Besides, time restrictions, not having the right mindset to explore topics openly, and reluctance to touch upon topics that seem (too) intimate, emotional, or personal have shown to be serious barriers.^10,11^ Lack of training has shown to be the most important barrier to spiritual care provision.^15–18^

Several studies described training courses to increase nurses’ communication competences regarding spiritual care, with varying results.^12,19^ Recurring elements in these interventions are multiday training sessions on communication skills and the use of roleplay or simulation patients to increase relatability and effectiveness. Moreover, integrated training approaches seem to have better results. No studies used workplace learning, also referred to as learning on the job, although reflecting on real cases from the work setting seemed to be successful in other studies.^20,21^ Until now, there is very little experience with this type of learning in oncology nursing or spiritual care training. For that reason, we aimed to bridge that gap by exploring the effects of meaning-centered coaching on the job of nurses in an oncology ward on their self-assessed spiritual care competences and job satisfaction, and the factors influencing it.

## METHODS

### Design

We used a mixed methods study design to understand whether coaching on the job affected oncology nurses’ spiritual care competences and job satisfaction, and the factors influencing it. We employed a participatory action research approach. Participatory action research involves a cyclical process of research, reflection, and action to improve an existing situation, based on input from relevant stakeholders.^22–24^ The theoretical framework underpinning the structure of the intervention consists of the work of Tynjälä^25^ on workplace learning. The literature and guidance in the Dutch national guideline *Existential and Spiritual Aspects of Palliative Care* guided the content of the intervention.^26^ Although patients were not necessarily in a palliative phase, facing a life-threatening disease triggers similar existential and spiritual questions and needs.^27^ Nolan’s definition around spirituality was guiding and perceived “as being hidden beneath, and often expressed in, the physical, psychological and social dimensions.”^26^ To learn to explore the spiritual dimension, often through these other dimensions, the intervention was centered on the assessment tool provided by the Mount Vernon Cancer Institute as described in the data collection.

### Participants

Participants involved an entire oncology nursing team, including the care manager and team leaders. All inpatient oncology ward nurses from an academic hospital in the Netherlands were invited to participate in the intervention. Inclusion criteria were employment on this ward, fluency in Dutch, and having provided informed consent. Using a purposive sampling technique, several of them were also invited by the team leader to participate in one or more of the 3 focus groups—in which 3 to 11 nurses took part—that were held throughout the intervention (see the Focus Groups section). We aimed for a diverse group in terms of age, work experience, gender, and affinity with the subject. Both the care manager and the team leader, with the latter also being one of the nurses, were approached for an individual interview at the start and end of the intervention.

### Data Collection

Data were collected through semistructured interviews, an online survey, focus groups, and team meetings. To answer the research questions, qualitative data (eg, regarding barriers and facilitators) stemming from different data-collection moments (eg, from focus groups and team meetings) were used interchangeably. See Table 1 for an overview.

**TABLE 1:** Overview of Data Collection

How?	What?	When?
Semistructured interviews with care manager and team leader (30-60 min)	- Envisioned and observed effects of the intervention - Expected barriers and facilitators	Before and after the intervention
An online survey filled out by participating nurses (10 min)	Quantitative intervention effects: a. Spiritual Care Competence Scale b. Job satisfaction (SWS subscale)	4 wk before and 4 wk after the intervention
Focus groups with participating nurses (30-60 min)	- Suggestions to optimize the intervention - Qualitative intervention effects (*reflectively*) - Experienced barriers and facilitators (*reflectively*)	One before, one during, and one after the intervention
Team meetings with participating nurses (30 min)	- Qualitative intervention effects (*observing*) - Experienced barriers and facilitators (*observing*)	Once a week during the intervention period

Abbreviation: SWS, Spiritual Well-Being Scale.

## SEMISTRUCTURED INTERVIEWS

As part of the participatory action research, both the care manager and team leader were interviewed individually before and after the intervention using semistructured interviews to respectively collect their envisioned and observed effects of the intervention. Besides, expected mediating factors (barriers and facilitators) potentially impacting the intervention and spiritual care provision were gathered. Along with the input from the focus groups, the obtained information guided the final design of the intervention. The interviews lasted 30 to 60 minutes and were audiotaped and transcribed verbatim. In addition, field notes were made during the interviews.

## SURVEY

To measure the effects of the intervention, participants filled out an online survey 4 weeks before and 4 weeks after the intervention (see Appendix I, available at http://links.lww.com/CN/A124). It is composed of the Spiritual Care Competence Scale (SCCS) and the job satisfaction subscale of the Spiritual Well-Being Scale.^28,29^ The SCCS assesses nurses' competences by measuring abilities on subscale assessment and implementation, professionalization and quality improvement, personal support and patient counseling, referral, attitude, and communication. It consists of 35 questions on a 5-point Likert scale ranging from “strongly disagree” to “strongly agree.” The Spiritual Well-Being Scale job satisfaction subscale contains 10 questions on a 10-point Likert scale ranging from “totally not” to “very much.”

## FOCUS GROUPS

In total, 3 focus groups were held: 1 before, 1 during, and 1 after the intervention in which nurses had the opportunity to reflect on the experienced effects of the intervention, on barriers and facilitators, and on the intervention process itself. Suggestions made to optimize the intervention were integrated where possible. The focus groups lasted 30 to 60 minutes and were audiotaped and transcribed verbatim.

## INTERVENTION (DURING NURSING STAFF TEAM MEETINGS)

The intervention consisted of weekly coaching on the job during the daily 30-minute nursing staff team meetings in which patients are discussed and took place from June to November 2021. In total, the coaching intervention took place in 18 of the 21 planned team meetings; 3 were canceled because of acute circumstances on the ward. Coaching focused on uncovering barriers and facilitators in providing spiritual care for both patients and themselves. The intervention was alternately conducted by 2 researchers with a background as counselor (L.M., J.v.M.). It centered around the following content: (1) in daily practice applying a modified version of the first Mount Vernon Cancer Network question (“What are you most occupied with at the moment?”) to explore what is meaningful to a patient,^30^ (2) learning to explore topics with patients instead of making own assumptions, (3) distinguishing between a patient’s values and own values, and (4) becoming aware of own experiences in taking care of patients. As suggested by nurses, at the beginning of the intervention, the Mount Vernon Cancer Network question was integrated into the electronic patient file. After the second focus group halfway through the intervention, a few changes were made to the coaching intervention. The most important adjustment, again suggested by nurses, concerned a recurring focus on positive aspects of caregiving as a counterbalance to the (perceived) difficulties. The team meetings were audiotaped, and parts of relevance for data analysis (regarding experiences' effects, and barriers and facilitators) were transcribed verbatim.

As nurses within a secular European setting like the Netherlands are primarily concerned with secular existential orientations such as meaning, value of life, and personal values that are not centered in religious ideological personal beliefs,^31^ the intervention had a meaning-centered approach. During the intervention, the word “spirituality” was replaced by “meaning” (“zingeving” in Dutch) when it concerned this dimension.

### Data Analysis

Quantitative data were analyzed using paired-sample *t* tests in SPSS version 25. For the SCCS and Spiritual Well-Being Scale job satisfaction subscales, general means were compared. For the SCCS, means on the 6 subscales were also compared. Qualitative data were analyzed using a conventional content approach.^32^ The transcripts of the first 3 team meetings were read and coded line-by-line by 2 researchers (L.M., A.W.) using Atlas.ti version 9. The codes were discussed until consensus about the codebook was achieved. The remaining team meetings and focus groups were analyzed by 1 researcher (L.M.) using the developed codebook. The first interviews were also coded line-by-line by 2 researchers (L.M., A.W.), and after consensus, they were finished by 1 researcher (L.M.). Two peer group sessions (L.M., A.W., Y.E., J.v.M.) took place to cluster codes and define categories and themes.

Finally, quantitative and qualitative findings were integrated and compared to better understand the effects of coaching on the job on nurses' competence and job satisfaction, and the barriers and facilitators influencing it.

### Ethical Considerations

Because participants were not subject to treatment or required to behave in a certain way, the Medical Research Ethics Committee Oost-Nederland concluded this study was not subject to the Medical Research Involving Human Subjects Act (2021-8158). Written (quantitative) or verbal (qualitative) informed consent was obtained from all participants. The anonymity of the participants and discussed patients was guaranteed by removing information from the transcripts that could lead to identification.

## RESULTS

In the description of the results, we distinguish between the effects of the intervention (quantitatively and qualitatively) and mediating factors facilitating or hindering providing spiritual care (qualitatively).

Of the 35 nurses invited, 3 left for a new job and 2 were on maternity leave during the intervention period. Consequently, 30 nurses were included at the start of the intervention. The unit-based design of the intervention meant that nurses participated in the intervention if it took place on their working day, regardless of whether they participated in the research through the survey and/or focus groups. An overview of the 28 participants who provided characteristics can be found in Table 2.

**TABLE 2:** Characteristics of Participants of the Intervention

Gender	Year of Birth	Highest Education	Function	Years of Experience
Male	1994	Secondary vocational education	Nurse	4
Female	1970	Higher vocational training	Nurse	31
Female	1989	Academic education	Case manager	10
Female	1991	Higher vocational training	Nurse	4.5
Female	1994	Higher vocational training	Nurse	3
Female	1959	Higher vocational training	Nurse	40
Female	2001	Secondary vocational education	Case manager	0
Female	1972	Higher vocational training	Nurse	20
Female	1992	Higher vocational training	Nurse	5
Female	1964	Higher vocational training	Case manager	37
Female	2000	Secondary vocational education	Nurse	1
Female	1990	Secondary vocational education	Nurse	8
Female	1961	Higher vocational training	Case manager	42
Female	1978	Higher vocational training	Nurse	15
Female	1995	Higher vocational training	Nurse	2
Female	1995	Higher vocational training	Nurse	3
Female	1997	Higher vocational training	Nurse	3
Female	1968	Higher vocational training	Nurse	7
Female	1994	Higher vocational training	Nurse	3
Female	1977	Higher vocational training	Nurse	20
Female	1996	Academic education	Nurse	3
Female	1996	Higher vocational training	Nurse	2
Female	1989	Higher vocational training	Nurse	12
Female	1964	Higher vocational training	Nurse	37
Female	1967	Higher vocational training	Nurse	40
Female	1991	Higher vocational training	Nurse	9
Female	1980	Higher vocational training	Nurse	5
Female	1988	Higher vocational training	Nurse	10

## THE EFFECTS OF COACHING ON THE JOB

### Envisioned Effects

In the interviews preceding the intervention, the manager and team leader articulated their expected increase in nurses’ job satisfaction and well-being when they would be facilitated in sharing and reflecting on spiritual care provision. These increased shared reflections were expected to be translated in better care for patients, contributing to high-quality person-centered patient care. This was understood as “tailored to a patient’s life and values, without judgment—and communicated to the physician if needed.” According to interviewees, this required an open attitude and proficient communication skills in spiritual care from nurses.

### Achieved Effects

#### Quantitative

The online survey response rate was 57% (n = 17). Self-assessed competence on the SCCS increased significantly (0.29, *P* < .001), especially on the subscales of *communication* (0.32, *P* < .01), *professionalization* (0.46, *P* < .01), and *personal support* (0.29, *P* < .05). No significant effects were found on job satisfaction (−0.01, *P* = .91) (see Table 3).

**TABLE 3:** Differences in Competence (SCCS) and Job Satisfaction (SWS)

	Pretest	Posttest	Significance (*P*)
SCCS (mean)	3.77	4.06	.00
Attitude	4.37	4.49	.48
Communication	4.29	4.62	.01
Assessment and implementation	3.95	4.11	.15
Referral	4.02	4.20	.12
Personal support and patient counseling	3.70	3.99	.02
Professionalization and quality improvement	3.16	3.61	.00
Job satisfaction subscale of SWS	8.04	8.03	.91

Abbreviations: SCCS, Spiritual Care Competence Scale; SWS, Spiritual Well-Being Scale.

#### Qualitative

The interviews, focus groups, and team meetings revealed 3 main categories of intervention effects, which are set out hereinafter.^a^

##### Becoming More Aware of Own Care Experiences

The first category is about nurses becoming more aware of how they experience caring for patients. Jointly sharing own experiences during team meetings—including accompanying questions and reflections—increased mutual connection and attention to spiritual care provision. Normalizing the topic lowered the threshold to discuss concerns or doubts in caregiving with fellow nurses or to address others on it.

I also notice during the team meetings, on Wednesdays but also on other days, that the nurse who brings in a case is asked how it is going or how it is for that person. I do notice a difference and I like that. But I found that with us anyway, there was often attention for that (ie, before the intervention); only then it was perhaps just asked if someone indicated it themselves or if you really noticed it in someone, and now it is perhaps asked a little earlier. (Nurse, focus group 2)

The intervention normalized not only the mutual conversation about spiritual care but also the perspective on how to work, as described by the team leader:

Nowadays, when someone stays with a patient longer, I don’t think “she’s cutting corners,” but rather: how good that she is taking the time. (Team leader, interview R2)

##### Exploring Without Judging

The increased focus on spiritual care helped in making nurses more aware of distinguishing their own values in life of those of patients. They began to realize that, in interactions with patients, they sometimes tend to unconsciously assume the patient has the same values as they themselves have. The coaching helped in seeing that, as nurses, their job is to explore the patient’s values in life and subsequent care wishes, without letting their own values and preferences guide them.

It’s more about being aware, that what I think about it is not of the utmost importance, that if it’s good for her [the patient], then it’s good. (Nurse, focus group 2)

In line with this, nurses described filling in less, to be more careful with assumptions about patients’ feelings. It was mentioned that they therefore now more often explored them, which they assumed leads to patients feeling more seen and heard.

So you have to keep that question [the Mount Vernon question: (“What are you most occupied with at the moment?”] in the back of your mind, I think it’s a very good one and patients like that too, because it gets them seen and heard, but not structured at a time. (Nurse, focus group 1)

##### Shifting Focus: What Goes Well?

Finally, the intervention led to an increased conscious focus on things going well in providing spiritual care. Nurses mentioned that the implementation of structural attention to this aspect in the second part of the intervention led to an increased perceived job satisfaction.

We never say positive things to each other, never is a big word, but few. And I think with positive energy you can also do quite a lot with your mindset and with your day. Of course, you shouldn’t give ten compliments every day, because then you get uncomfortable, but if someone gets a compliment once a week like “you worked so well today, you did so well for the patient,” then I think yes, that’s right, and then I’m proud of that and then you feel much better about yourself. (Nurse, focus group 2)

The care manager and team leader noticed an increased mutual connection between the nursing staff. More attention was being paid to their own well-being, as well as the well-being of their colleagues.

I notice that the team leaders come to me more often to say that we need to pay close attention to someone, that there is more care towards each other. (Care manager, interview R1)

## Facilitators and Barriers in Providing Spiritual Care

The interviews, focus groups, and team meetings also revealed barriers and facilitators mediating intervention effects in providing spiritual care. An overview of the barriers and facilitators identified was compiled in the 3 categories *nurses’ attitudes*, *support structures*, and *professional relations* (see Table 4).

**TABLE 4:** Facilitators and Barriers in Providing Spiritual Care

	Facilitators	Barriers
1. Nurses’ attitudes	a. Sensitivity and responsiveness to patient needs b. Having time to explore c. Diverse approaches in the team d. Care vision and word use	e. Busyness and practical incline f. Nurses’ unawareness of own values g. Making assumptions about what patients think and feel
2. Support structures	a. Continuation of coaching b. Regular coaching c. Integration of attention to spiritual care in work structure	d. Terminology (eg, negative associations with the term *spirituality*) e. Imposing intervention f. Lack of integration to spiritual care in history taking and transfer
3. Professional relations	a. Supporting team leaders b. Supporting colleagues c. External coaching d. Open attitude patient	e. Hierarchical relation with physicians f. Large group

### Nurses’ Attitudes

Nurses’ attitudes include nurses’ attitudes and habits that were mentioned as aspects promoting or hindering their spiritual care provision.

#### Facilitators

Effectively identifying and exploring what is meaningful to patients showed to be dependent on the nurses’ attitude: (1) *being sensitive and responsive to patients’ needs* regarding what is of meaning to them. Some nurses expressed the necessity to distinguish between the patients’ need to talk about meaning-related themes and their own need to address them. Providing spiritual care should match a patient’s wish and be explored at an appropriate time.

I also think that sometimes the person doesn’t always know what to do with the question [the Mount Vernon question: “what are you most occupied with at the moment?”]. So I don’t ask the question, because I don’t know what to do next. And of course, some nurses ask this question all the time, and sometimes patients literally say: well, this nurse doesn’t need to ask this question today. It is also a degree of appropriateness, this is very much putting it in boxes, of whether something is appropriate or not. But it has to do with feeling, being sensitive. (Nurse, focus group 2)

Having the opportunity to collect pieces of the puzzle during a patient’s hospital stay—(2) *having time to explore*—instead of in 1 conversation, was considered helpful. Moreover, it was a positive to be able to explore meaning-related topics in a way and at a time convenient to himself/herself. Reluctance to discuss these topics decreased when nurses were given the autonomy to make it their own—without having to conform to a certain protocol.

Most nurses appreciated the (3) *diverse ways of approaching meaning-related topics in their team*. They perceived such different approaches as complementary, together enhancing the quality of patient care:

But I think that is also the beauty of it, that you can complement each other. Suppose N4 is on day shift and just had a nice deep conversation and N1 is on evening shift and she brings in some lightness, then there is a balance. You can’t just have tough conversations all day long. (Nurse, focus group 2)

The nurses’ (4) *care vision* influenced their attention to spiritual care. However, sometimes the terminology or *word use* related to spiritual care created challenges in communication between nurses because it evokes (negative) stereotypical associations. Moreover, although some nurses do not explicitly discuss meaning-related issues, they were praised for their holistic approach and are known to (unconsciously) focus on providing spiritual care.

They think these questions are too spiritual, too “woo woo.” Yes, but we do talk about it and it does come up and then it needs to have a name again. I think that some nurses really do talk about it and they don’t feel it is spiritual care, they just see it as part of their work without it having a name. (Team leader [nurse], interview R1)

#### Barriers

One of the most frequently mentioned challenges when it comes to the nurses’ attitude was their (5) *busyness and practical incline*. Solving problems instead of taking the time to explore the actual question often was the default mode.

I think that as nurses we are very practical-oriented and trained. [...] So I would say that that’s what you go on about, what you can solve and what you can do at that moment. I also notice this in rounds, when we bring in a new patient and he has questions about school, we are the first ones to say “oh we can ask social work and we can do this and do that,” ok solved. While sometimes we could also dwell a bit more on oh gosh, he has questions about school, but what is it that keeps him busy about school? Is he afraid that he cannot go to school? That he cannot finish his education? There’s also an underlayer under the fact that this boy would like to ask something or is worried about school. And I think sometimes we can be a little more aware of that. But that’s just what happens because that’s just what you can tick off at that time. (Nurse, focus group 2)

In line with this, the expected time investment necessary to explore meaning-related topics prohibited nurses to engage in conversations regarding this dimension with patients, often unconsciously. At times, they also shied away from such conversations, because they were perceived as uncomfortable or intense:

I’m not really the best example I think. I do like humor and a certain lightness, and I think that is also partly self-protection, because every time such a deep conversation takes place, that also affects me. For me, it shouldn’t become too intense. (Nurse, focus group 1)

Whereas their busyness and practical incline were familiar to them and often referred to, the limiting attitude that comes from (6) *being unaware of their own values* was surprising to the nurses. Nurses, sometimes, wrongly assumed patients value the same things in life as they do.

This realization made nurses aware that they (7) *make assumptions about what patients think and feel*, without exploring them. This regularly led to guesswork in the team meetings about a patient’s behavior or wishes. The coaching then centered around becoming aware of the limiting effect of this way of working and solving it by going back to the patient to explore the topics that came up:

When it came to what quality of life was for the patient, I noticed that, not that you fill in the blanks yourself, but maybe you subconsciously think of your own things as important. I didn’t ask it specifically while it was actually running through the patient’s whole day. You [the coaches] can just ask the right questions so that you are triggered to think a bit more about it with the patient. (Nurse 1)

You give your own opinion about it, but for someone else quality can be something completely different, but then it’s hard to let go of yourself a bit because sometimes you think: “is that quality for you?” It’s hard for me to imagine so to speak and you’re going to give your own quality the upper hand over what the patient would want. I don’t want that, but it does happen. (Nurse 2)

And then it’s nice that someone else asks you at a certain point, because then you notice, oh, I don’t really know [what the patient values]. (Nurse 1, focus group 2)

### Support Structures

Support structures are structures or processes in a department to streamline care delivery, such as team meetings or reporting systems. These support structures can either increase or prevent attention to spiritual care provision.

#### Facilitators

(1) *Continuation of the coaching* was mentioned as one of the most important facilitating factors. This goes for the actual intervention period but was also mentioned as one of the essential components for creating sustainable future attention for this part of patient care.

Yes, because you do something structured and more often, there is structure again and otherwise it is a bit strange to suddenly ask a blank question which is normally never done. And now it’s more normal and yes, I do like it. (Nurse, focus group 2)

At least when I look at when one of the coaches was around, we also looked at ourselves a bit more. What does it do with you as a team and what happens to you when you’re there? And I had the feeling when spiritual care wasn’t around, that it was becoming a bit diluted. (Nurse, focus group 2)

(2) *Regular coaching*—at least weekly—of the nurses was appreciated as a way to pay attention to the nurses themselves as well as help them increase their ability to provide spiritual care. Looking at the future, a wish was expressed for increasing the coaching to 2 times a week to reach more nurses who often work on different days, because the presence of a coach makes a difference.

Moreover, (3) *integration of attention to spiritual care into the existing work structure* proved to be one of the crucial elements for successful implementation. Not only did it work smoothly, it also provided several reminders throughout the day to pay attention to this dimension. Integration into the existing work structure consisted of a reminder at the start of the day by the nurse in charge to ask the Mount Vernon question, including feedback on that exploration during the doctor’s visit halfway through the morning, discussing it with colleagues after lunch, and, at the end of the day, reporting it in the electronic medical record where the Mount Vernon question was inserted in the format instead of the term “spiritual dimension.” This last practical adjustment proved to be especially helpful:

I think it does add value, especially that question in the electronic medical record. And naming it, especially if you come on a fixed day and it’s in the index card every day, so you also mention under the heading “spiritual care” or something like that you come and if you then see that specific question every time, I think that’s a good reminder that, oh yes, I have to pay attention to it. (Nurse, focus group 1)

#### Barriers

When it comes to barriers, (4) *terminology—and particularly negative associations with the term spirituality—*was mentioned.

Yes, for me that does provide clarity [using the Mount Vernon question], because then you just have a question and you know more or less what to ask, not literally, but which way you can go, and you can also answer it more easily, “the patient is most concerned with....” Because “spirituality”—I personally find it a very heavy word with which I have certain associations. (Nurse, focus group 1)

Getting nurses on board to increase their attention to spiritual care is (5) *expected to fail when imposed on them*. Cultivating intrinsic motivation and giving it time to grow on them is proposed as the preferred strategy as this quote shows:

And you have to dress it up a bit nicely that it’s part of the care and not because you find it interesting yourself. Because in the standard electronic patient file, there is a heading for spirituality that was used a while ago: “We need to pay more attention to spirituality,” but nobody fills in the heading because it is imposed, you know? There must be an intrinsic motivation so that you are fascinated and touched instead of doing this now because it is said to be a quality improvement. Every week someone comes to the hospital with a new plan, but yes, you have to be really triggered. (Nurse, focus group 1)

(6) *Not integrating attention to what is meaningful to a patient in history taking or transfer* to other healthcare providers is perceived as a barrier as well:

No, we don’t have an electronic medical record-focused format or a SMART text on how to transfer to home care or anything like that. Team leader, interview R1)

### Professional Relations

Professional relations include all interactions between healthcare providers in the nursing department related to patient care. These relationships can promote or hinder attention to spiritual care in various ways.

#### Facilitators

Facilitating factors were mentioned related to the team, the coach, and the relationship with the patient. When it comes to the team, it was expressed that (1) *support by the team leaders* to address spiritual care was helpful. (2) *Supporting colleagues*, that is, helping each other out by taking over care tasks for a colleague to give him/her more time to invest in meaning-centered conversations or care, proved to be supportive and appreciated:

At the time of the patient’s passing, I was there for her loved ones. I was not really called away, because there were two of us, so I could say to N2 “you finish room 28, and I will stay here with the family.” So I was able to help them all along, we were able to wash her together, we were able to take our time, we all went to the mortuary together, that’s nice, that that’s possible. And N3 took my pager for a while, I’ve had my phone off for a while, that that’s possible, that for me makes that I had the time to be able to give the care that I wanted to give. (Nurse, team meeting 16)

Moreover, (3) *having an external coach specialized in spiritual care* was felt to help address meaningful topics. The coach’s attitude was specified as respecting the nurses’ profession, supporting the conversation instead of taking over and making a heartfelt connection:

You focused on “what does that do to you?” You (ie, the coach) don’t take over, you connect to where we are. What you do is sit back and listen. In our work, the emphasis is on clinical reasoning, you don’t do that. The attitude of sitting back is opposite to that and thus complements it. It is a different way of communicating and making contact. It’s about connecting, also with the perception of the nurse. Real contact, paying attention to each other. (Care manager, final interview R2)

Finally, (4) *an open attitude of the patient* was perceived as helpful (or hindering when framed negatively) in having a meaning-centered conversation, as this nurse shares:

Of course, some patients are easier and more open than others and with some you have a nicer connection to sit down with than others. If you have someone who very much resists you, then it is of course much more difficult, but if you have someone with whom you can chat all day, then asking such a question is much easier. That all plays into it as well. (Nurse, focus group 2)

#### Barriers

The (5) *hierarchical relationship with the physicians* by some nurses was perceived as challenging in addressing spiritual care. Sometimes the perceived responsibilities of a physician by a nurse led to unhelpful assumptions:

I think sometimes we should listen better to our patients, and not just the nurse but certainly the doctors should. They, of course, have taken an oath to treat, up to, well, and we may have earlier that we think: should we do this? And that we then have to enter into a conversation with the patient to ask “where are you now?”, “is this really what you want, otherwise you have to discuss this with the doctor.” And then you’re also dealing with a bit of a hierarchy. (Team leader, interview R2)

Some nurses felt uncomfortable sharing their challenges in (6) *a larger group of nurses*. Especially when a significant part of the group consisted of nurses in training, some nurses refrained from sharing their experiences, as this example shows:

For example, today there were twelve of us in the coffee room with ten students, so I don’t show the back of my tongue about what this has done to me, I am very honest about that. But I also remember that the mother of a patient really raged against me and that I walked up to the team leader and thought “what is happening to me now?” And then the team leader can very well ask the questions and then it comes out, but if I am sitting with ten of those people then I would also just keep my mouth shut. So that is a tricky thing, what is a good time for that? (Nurse, focus group 2)

## DISCUSSION

This study explored the effects of coaching on the job of oncology nurses. A significant overall increase in their spiritual care competences was found, but no impact on job satisfaction.

Specifically, competences in *communication*, *personal support*, and *professionalization* increased. Nurses described an increased awareness of their own experience of taking care of patients and a developed ability to distinguish their own values from those of the patient. The intervention led to normalizing discussions between nurses on what matters most for patients as well as a conscious focus on the actual care provided to patients. Several mediating factors were identified influencing spiritual care provision, which could be categorized in the nurses’ attitudes, support structures, and professional relations.

### Comparison With Previous Literature

One of our key findings is that nurses had difficulty recognizing their own values and differentiating them from those of the patients. This confirms a study showing that addressing healthcare providers’ personal values and meaning is the strongest factor to improve spiritual care provision.^33^ Training should therefore not be limited to providing patient-centered care but also include attention to the providers’ experience. One of the biggest challenges we found was related to work attitude, and that it is needed to move from a place of “busyness” to working attentively and reflecting on the care provided, an aspect also described in other studies.^13,34,35^ The “culture of busyness” is an attitude most nurses learn from the start of their working career and emphasizes task orientation, which resembles the traditional working culture in healthcare. Learning to pause and reflect requires a supportive work culture, optimal physical and work structures, the support of management, and good personal relations.^36^

Moreover, this study shows the unease nurses felt with the term *spirituality*. The word does not appeal to them, and although often used in the medical field, it is associated with “heavy” and “woo-woo” topics. This was also found in adjacent articles.^37–40^ Not surprising, given that medical dictionary definitions of spirituality, for example, are “an awareness of the metaphysical, the religious, or the sublime”^41^ and “something that in ecclesiastical law belongs to the church or to a cleric as such” or “sensitivity or attachment to religious values.”^42^ Therefore, the coaching on the job of this study was meaning centered, and the word *spirituality* was not used during the intervention.

No quantitatively measured changes in job satisfaction were found; however, with an 8/10, this was already quite high at baseline. This is not to say it was not affected. One of the biggest changes made based on nurses’ requests after the midway evaluation was an increased focus on the positive side of their work and performance. This need for approval and support from peers and team leaders is in line with research on job satisfaction among millennial nurses, which made up a large part of the nursing team in our study.^43^

Study results are in line with insights on effective workplace learning. Essential characteristics used are collaboration, the use of various tools as opposed to solely cognitive reasoning, applying concrete case studies, and learning tailored to the situation.^25,44,45^ By using the existing work structures for workplace coaching on the job, necessary infrastructure was put in place to support learning. Doing this ensures learning is embedded in the organization, especially relevant given the large turnover of nurses.^46^ In this study, integration of the Mount Vernon question (*What are you most occupied with at the moment?*) into the electronic patient record proved to be an important catalyst.

### Strengths and Limitations

This study yields clinically relevant findings to move the field forward. Moreover, the participatory action research study was performed with the involvement of an entire oncology nursing team, including the care manager and team leaders. This co-creative approach proved valuable amidst a demanding COVID-19 pandemic. However, our study also has limitations. First, the main researcher combined performing the coaching process and the interviews, which might have caused bias. We minimized this risk by working with another coach throughout the study and by analyzing data with 2 independent researchers. Second, this study was executed during a COVID-19 wave. Many nurses were working overtime and on COVID-19 wards to collectively bear the burden of the pandemic. As a result, questionnaire response rates were relatively low. Finally, we implemented the intervention at the team level but measured effects at the individual level. All nurses were exposed to the intervention, but not all of them completed the survey. Although we also expect effects on nonresponders given the unit-based design of the intervention, we are not able to confirm this hypothesis.

### Implications for Practice and Future Research

Attention to and improving spiritual care competences should be integrated into existing work structures, and the terminology used should match common understandings and sentiments. Coaching nurses on the job can increase self-assessed spiritual care competences—assessing whether this also translates into improved quality of care and communication could be the next step.

## CONCLUSION

Coaching oncology nurses on the job increased spiritual care competences. No effects on job satisfaction were found. Mediating factors could be categorized in nurses’ attitudes, support structures, and professional relations. Workplace learning proved to be an effective learning strategy, because of the involvement of nurses in the development of the training program and favorable conditions such as workplace culture, integration in existing work structures, management support, and good personal relations.

## Supplementary Material

## Footnotes

^a^
As the interviews and focus group had a more reflective nature, and the team meetings had a more observing nature, most illustrative quotes were derived from the former.
